# IR780 Based Sonotherapeutic Nanoparticles to Combat Multidrug-Resistant Bacterial Infections

**DOI:** 10.3389/fchem.2022.840598

**Published:** 2022-01-24

**Authors:** Biying Huang, Long Wang, Kui Tang, Sijie Chen, Yan Xu, Haiqin Liao, Chengcheng Niu

**Affiliations:** ^1^ Department of Ultrasound Diagnosis, The Second Xiangya Hospital, Central South University, Changsha, China; ^2^ Research Center of Ultrasonography, The Second Xiangya Hospital, Central South University, Changsha, China; ^3^ Department of Orthopedics, Xiangya Hospital, Central South University, Changsha, China; ^4^ Hunan Engineering Research Center of Biomedical Metal and Ceramic Implants, Xiangya Hospital, Central South University, Changsha, China; ^5^ National Clinical Research Center for Geriatric Disorders, Xiangya Hospital, Central South University, Changsha, China; ^6^ Hunan Key Laboratary of Aging Biology, Xiangya Hospital, Central South University, Changsha, China

**Keywords:** IR780, sonodynamic therapy, multidrug-resistant bacterial infections, antibacterial therapy, reactive oxygen species

## Abstract

Multidrug-resistant (MDR) bacterial strains have emerged and weakened the therapeutic effects of antibacterial drugs. Sonodynamic therapy (SDT) takes advantage of noninvasiveness and deep tissue-penetrating features and has been rejuvenated to combat MDR bacteria and their biofilm-associated infections. To improve the efficacy of antibacterial SDT, we first developed IR780-based PLGA nanoparticles as sonosensitizers for high-frequency ultrasound (US)-monitored antibacterial SDT of MRSA myositis by therapeutic low-frequency US. In this study, the developed shell-core-structured IR780@PLGA nanoparticles were designed with a polymer shell PLGA with the sonosensitizer IR780 loaded on. High-frequency diagnostic US was introduced to monitor the sonotherapeutic progression of bacterial myositis by therapeutic low-frequency US. Importantly, the *in vitro* and *in vivo* results confirmed that IR780@PLGA nanoparticles combined with US irradiation possess high efficiency for antibacterial therapy. This approach provides a simple and efficient strategy to monitor and combat MDR bacterial infection.

## Introduction

Bacterial infection is one of the most serious health problems worldwide. Due to the overuse and misuse of antibiotics, multidrug-resistant (MDR) bacterial strains have emerged and weakened the therapeutic effects of antibacterial drugs (Jia et al., 2019). Antibiotic-free antibacterial techniques based on “nondrug” sterilization methods have attracted increasing attention (Wei et al., 2019). Photodynamic therapy (PDT), photothermal therapy (PTT), and sonodynamic therapy (SDT) have been rejuvenated to combat MDR bacteria and their biofilm-associated infections (Pang et al., 2019a; Hong et al., 2019).

Antibacterial SDT relies on the ability of low-frequency ultrasound (US) to activate a sonosensitizer and trigger the generation of reactive oxygen species (ROS) to achieve high lethality for virtually all bacteria against MDR bacterial infections (Serpe and Giuntini, 2015). Compared to PDT- or PTT-induced antibacterial therapies limited to skin lesions, SDT takes advantage of noninvasiveness and deep tissue-penetrating features, showing great potential in deeply seated infections, and less systemic toxicity (Pang et al., 2019b; Wang et al., 2021).

**FIGURE 1:**
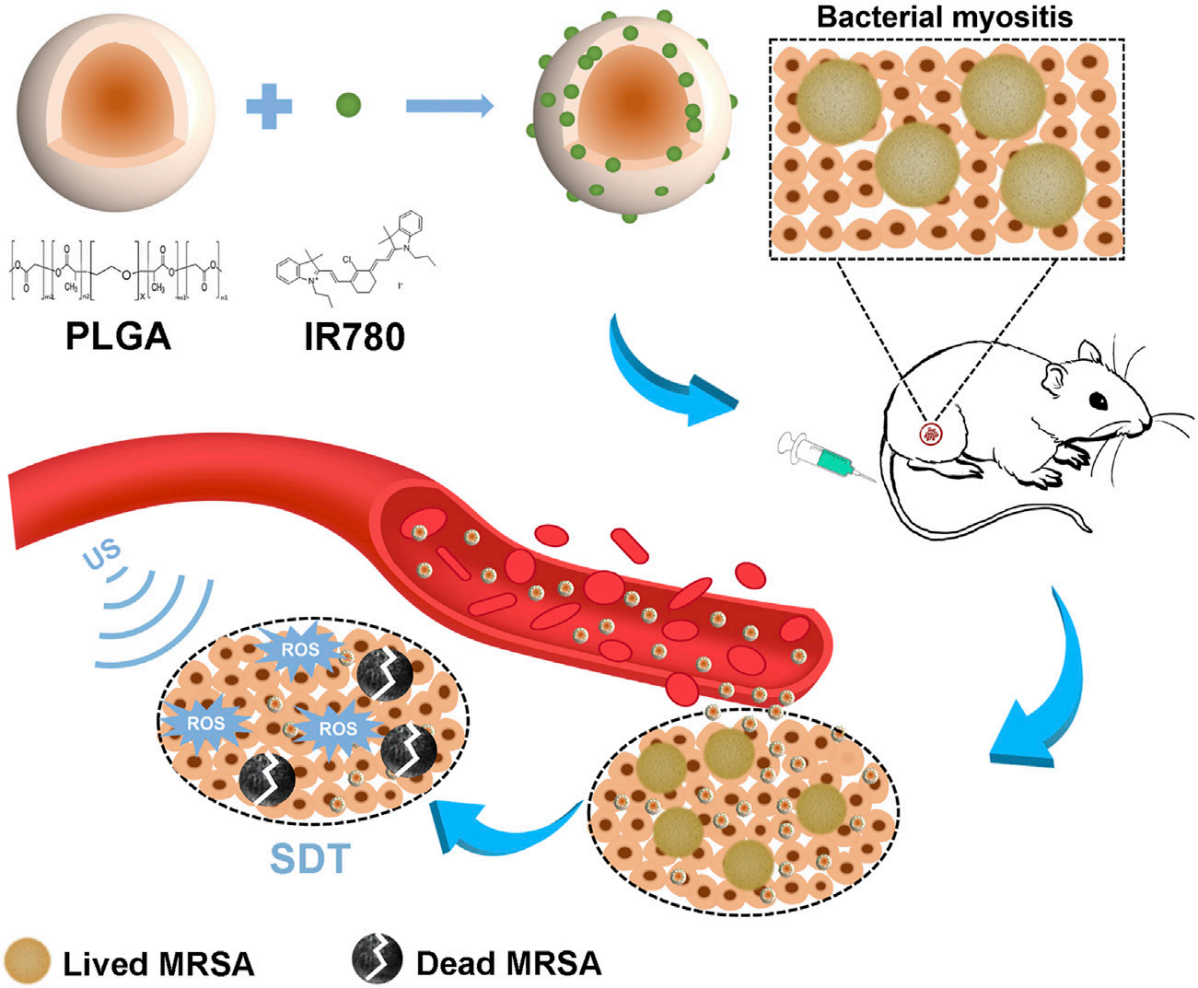
Schematic illustration of the sonotherapeutic IR780@PLGA nanoparticles for combating multidrug-resistant bacterial infections.

IR780, a small molecule cyanine dye with a strong optical property and excellent photoconversion efficiency following near infrared (NIR) irradiation, has attracted increasing attention in the field of cancer phototherapy (Wang and Niu, 2021). IR780 can emit fluorescence with a high intensity, long retention time and deep penetration within the NIR region in tissue, which enables its wide utilization for NIR fluorescence (NIRF) and photoacoustic (PA) imaging applications (Yang et al., 2020). As a photosensitizer, it was used to produce single oxygen to enhance the susceptibility of the bacteria to heat and increase the bacterial cell membrane permeability (Wang X et al., 2018; Tan et al., 2018). As a sonosensitizer, upon US irradiation, the generation of ROS by IR780 demonstrated a significant increase of ^1^O_2_ level and H_2_O, but not ⋅OH in the SDT-treated cells (Li et al., 2016). For antitumor SDT therapy, IR780-based nanomaterials were proven to be highly effective in promoting reactive oxygen species generation and inducing cancer cell death (Qian et al., 2016; Chang et al., 2019; Wu et al., 2019; Zhang et al., 2019). However, few studies have reported IR780 as a sonosensitizer to combat MDR bacterial infection. Our previous study showed that sonosensitizer IR780-based and oxygen-sufficient drug-loaded nanoparticles can reverse the hypoxic tumor microenvironment and improve the efficacy of chemosonodynamic antitumor therapy (Huang et al., 2020). In this study, we first reported IR780-based PLGA nanoparticles (IR780@PLGA) for antibacterial SDT of methicillin-resistant *Staphylococcus aureus* (MRSA) myositis. Diagnostic US has a noninvasive, inexpensive, and fast detection nature, and this high-frequency portable US can be brought into the animal room to monitor the inflammation progression of mice more accurately in the barrier system to reduce the limitation that animals have to be brought out of the animal room for monitoring by other methods. For *in situ* visualization of antibacterial SDT, high-frequency US was introduced to monitor the sonotherapeutic progression of IR780@PLGA in mice with bacterial myositis (Figure 1). Thus, IR780@PLGA can be applied as a useful antibacterial SDT sonosensitizer for enhancing antibacterial efficacy against MDR bacterial infection.

## Materials and Methods

### Materials

IR780 iodide, PLGA, and polyvinyl alcohol (PVA) were purchased from Sigma-Aldrich (United States). A DCFH-DA Reactive Oxygen Species assay kit was purchased from Beyotime Biotechnology (China), and the Singlet Oxygen Sensor Green (SOSG) probe was provided by Thermo Fisher (United States). Other reagents were of analytical purity and were used without further purification. *Staphylococcus aureus* strains (MRSA, ATCC43300) involved in the study were obtained from The Second Xiangya Hospital of Central South University (Changsha, China).

### Preparation of IR780@PLGA Nanoparticles

The IR780@PLGA nanoparticles were prepared by a single emulsion evaporation method based on our group’s previous studies (Wang L et al., 2018; Chen et al., 2020; Huang et al., 2020). In brief, 50 mg of PLGA and 1 mg of IR780 were completely dissolved in 2 ml of chloroform. Then, 10 ml of 4% w/v PVA solution was added to the PLGA solution and emulsified for 2 min with an ultrasonic processor (Sonic, VCX150, United States). The resulting emulsion was mixed in 20 ml of deionized water and stirred for 3 h. Next, the resulting NPs were washed with deionized water (10 000 rpm, 20 min) until the supernatant became colorless. All procedures were performed in the dark.

### Characterizations

The morphology of the IR780@PLGA nanoparticles was observed by transmission electron microscopy (TEM, Jem-1400 Plus). The size distribution and zeta potential were analyzed using a Malvern size analyzer (ZEN3600, Malvern Instruments, US). The presence of IR780 in the NPs was verified on a UV–Vis–NIR spectrophotometer (UV-2450, Shimadzu, Japan). The encapsulation and loading efficiencies of IR780 were measured according to previous studies (Wang L et al., 2018; Chen et al., 2020). The production of ^1^O_2_ was measured using SOSG as a fluorescent probe. The ultrasound (US) irradiation times (1 MHz, 2 W/cm^2^) using a US transducer (WED-100, WELL.D Medical Electronics, China) were 0, 30, 60, 90, 120, 150, 180, and 210 s. The fluorescence spectra of SOSG were acquired on a fluorescence spectrometer with an excitation wavelength of 504 nm. Stability experiments of IR780@PLGA nanoparticles were measured in 1 × PBS or in 10% fetal bovine serum (FBS) using a Malvern size analyzer over 7 days.

### *In Vitro* Antibacterial Activity Assay

The MRSA ATCC43300 strains were cultured in the tryptic soy broth (TSB) culture medium containing chloramphenicol at 10 μg/ml. To evaluate the dose-dependent antibacterial capability of IR780@PLGA nanoparticles, 100 μl of MRSA bacteria (10^6^ CFU/ml) was incubated with different concentrations of IR780@PLGA nanoparticles (2.5, 5.0, and 10.0 mg/ml, each 100 μl). The group without IR780@PLGA nanoparticles treatment was used as a control. After 2 h of incubation at 37°C with shaking, aliquots from the suspensions were serially diluted 10 times, and 20 μl of diluted bacterial solutions were plated on TSB agar plates. The number of bacterial colonies was counted after culture at 37°C for 24 h, and each sample was prepared in triplicate.

To evaluate the US intensity–dependent antibacterial capability, MRSA bacteria (10^6^ CFU/ml) were introduced to different intensities (0.5, 1.0, 1.5, and 2.0 W/cm^2^) of interval US irradiation (1 MHz, 30 s on and 30 s off for four on/off cycles). Then, bacterial colonies treated with US were counted following the aforementioned procedures.

To evaluate the SDT therapy of IR780@PLGA nanoparticles, 100 μL of MRSA bacteria (10^6^ CFU/ml) was incubated with the same concentration of IR780@PLGA nanoparticles (10.0 mg/ml, each 100 μl) and then introduced to different intensities (0.5, 1.0, 1.5, and 2.0 W/cm^2^) of interval US irradiation (1 MHz, 30 s on and 30 s off for four on/off cycles). After 2 h of incubation at 37°C with shaking, aliquots from the suspensions were serially diluted 10 times, and 20 μl of diluted bacterial solutions were plated on TSB agar plates. Then, bacterial colonies treated with US were counted following the aforementioned procedures.

Overnight-cultured MRSA bacteria (5 × 10^7^ CFU/ml, each 100 μl) were suspended in fresh TSB broth containing 1.0% glucose (w/v) with different treatments to form biofilms at 37°C for 36 h in 96-well plates. The groups were randomly divided into four groups: 1) PBS, 2) only ultrasound irradiation (US group, 2 W/cm^2^), 3) only IR780@PLGA nanoparticles (10 mg/ml), and 4) IR780@PLGA nanoparticles (10 mg/ml) with ultrasound irradiation (IR780@PLGA + US group). After different treatments in each group, the supernatant in the wells was discarded and rinsed with PBS, and then the wells were heated in hot air at 60°C for 60 min and fixed with methanol for 10 min. Finally, the biofilm was stained with 2.0% (w/v) crystal violet staining solution for 15 min and quantified by dissolving the dye in absolute alcohol and detecting the absorbance at OD 570 nm.

### Bacterial ^1^O_2_ Detection

MRSA bacteria (5 × 10^6^ CFU/ml) were divided into four groups as described previously: 1) PBS group, 2) US group, 3) IR780@PLGA group, and 4) IR780@PLGA + US group. MRSA bacteria were washed and incubated with 2ʹ,7ʹ-dichlorofluorescin diacetate (DCFH-DA, 10 μM) for 40 min to detect ^1^O_2_ generation and were then irradiated by ultrasound (1 MHz, 30 s on and 30 s off for four on/off cycles) in groups 2 and 4. For ^1^O_2_ detection, DCFH-DA has no fluorescence, while ^1^O_2_ generation can oxidize it to produce 2.7-dichlorofluorescein (DCF) with green fluorescence (Chen et al., 2020). Then, the bacteria were washed three times by centrifugation (4°C, 6,000 rpm) and observed by fluorescence microscopy.

### Morphological Study of Bacteria

A biofilm of MRSA bacteria was obtained following the previously described method and fixed with 2.5% glutaraldehyde at 4°C. Then, biofilms were dehydrated in ethanol, coated with osmium, and observed by scanning electron microscopy (SEM, JSM-7800F, JEOL, Japan).

### Animal Model

Female Kunming mice (6 weeks old, 25 g) were obtained from the Medical Experimental Animal Center of The Second Xiangya Hospital, Central South University (Changsha, China). All animal experiments were approved by the Ethics Committee of The Second Xiangya Hospital of Central South University and conducted in accordance with the guidelines of the Department of Laboratory Animals of Central South University. The right thighs of the mice were shaved and intramuscularly injected with 50 μl of MRSA bacterial solution (1 × 10^9^ CFU/ml). After 3 days, the thighs of the mice showed skin redness and swelling, indicating that bacterial infection models were successfully established.

### *In Vivo* Imaging of Bacterial Infections

For *in vivo* fluorescence imaging, a Lumina IVIS Spectrum imaging system (PerkinElmer, United States) was set up to acquire the fluorescence images. Bacterially infected mice were intravenously injected with 200 μl of IR780@PLGA nanoparticles (10 mg/ml) and imaged *in vivo* at different time points (8 h preinjection and 24 h postinjection). Then, the legs and major organs were harvested and imaged for *ex vivo* fluorescence. The average fluorescence intensities were calculated using the IVIS spectrum imaging system.

### *In Vivo* Antibacterial SDT of MRSA Infections

To evaluate the antibacterial SDT efficacy of IR780@PLGA nanoparticles, bacterial-infected mice were divided into four groups (*n* = 5): 1) saline group, 2) US group, 3) IR780@PLGA group, and 4) IR780@PLGA + US group. The mice in groups 3 and 4 were intravenously injected with 200 µl of IR780@PLGA nanoparticles (10 mg/ml). The mice in groups 2 and 4 were exposed to US irradiation (1 MHz, 2.0 W/cm^2^, 30 s on and 30 s off for four on/off cycles, 4 min in total). The mice in group 4 received the nanoparticles injection first and 24 h later exposed to US irradiation. To further assess the treatments in visualization, the ultrasound images of the legs were acquired on a portable US apparatus Vinno 8 (VINNO, Suzhou, China) at different time points (0, 4, 9, and 14 days), and the body weights were also measured at these time points. This high-frequency US apparatus provided B- and CDFI-mode US images. After different treatments at 14 days, the legs were harvested and stained with hematoxylin and eosin (H&E).

### Serum Biochemical Analysis

For assessment of the biological toxicity of the IR780@PLGA nanoparticles, twelve healthy female Kunming mice were intravenously injected with 200 μl of 10 mg/ml IR780@PLGA nanoparticles. Another three healthy mice without injection of nanoparticles were used as the preinjection of the nanoparticles group. The blood samples to evaluate hepatic functional markers, alanine aminotransferase (ALT) and aspartate aminotransferase (AST), and the renal functional markers, urea nitrogen (UREA) and creatinine (CREA), were collected for serum biochemistry assays at 3, 7, 14, and 28 days after nanoparticles injection.

### Histological Analysis

To further evaluate the biological toxicity of the IR780@PLGA nanoparticles in mice with MRSA infections, twelve female Kunming mice with MRSA infections were intravenously injected with 200 μl of 10 mg/ml IR780@PLGA nanoparticles. After 28 days, the mice were sacrificed, and the major organs, including the heart, liver, spleen, kidney, and lung, were harvested for H&E staining.

### Statistical Analysis

All results are shown as the mean values ± standard deviations. One-way ANOVA and Student’s t-test were adapted to analyze the data using SPSS version 21.0 software (IBM, Armonk, NY, United States). *p < 0.05 was considered statistically significant.

## Results and Discussion

### Preparation and Characterization of IR780@PLGA

**FIGURE 2:**
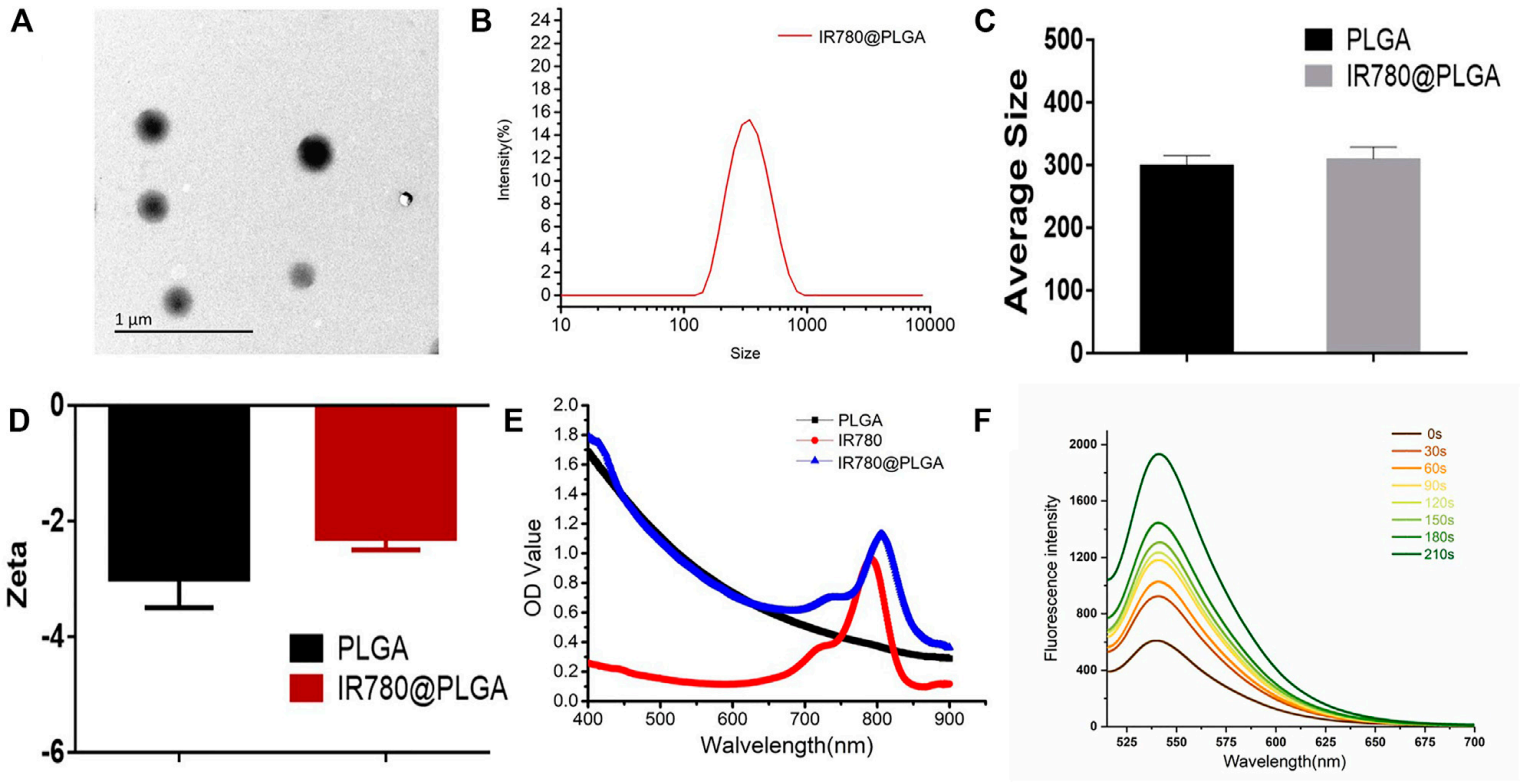
**(A)** TEM image of IR780@PLGA nanoparticles (scale bar = 1 μm). **(B)** Size intensity distribution of the IR780@PLGA nanoparticles. **(C, D)** Average diameters and surface zeta potential of PLGA and IR780@PLGA nanoparticles. **(E)** UV-Vis-NIR absorption spectra of PLGA, IR780, and IR780@PLGA nanoparticles. **(F)** Time-dependent ^1^O_2_ generation of IR780@PLGA nanoparticles using SOSG as a fluorescence probe with US irradiation (1 MHz, 2.0 W/cm^2^).

The IR780@PLGA nanoparticles were prepared by a single emulsion evaporation method. To observe the surface of the IR780@PLGA nanoparticles, the TEM image showed that the IR780@PLGA nanoparticles were smooth and spherical, with an average size of 300 nm (Figure 2A). The size intensity distribution and zeta potential of the IR780@PLGA nanoparticles were tested using a Malvern size analyzer. The average size of IR780@PLGA from the Malvern size analysis was 310 nm, and the surface charge was −3.0 mV (Figures 2B–D). However, the average size of the PLGA nanoparticles was 300 nm, and the surface charge was -5.0 mV (Figures 2C,D), meaning that the size and surface charge of the PLGA nanoparticles were not significantly different after loading IR780 onto the shell of the PLGA nanoparticles. To investigate IR780 loading, the UV–Vis–NIR spectrum of the IR780@PLGA nanoparticles showed an absorption peak at 780–790 nm, which was in line with the peak absorption of free IR780 with only a slight redshift, indicating the successful loading of IR780 (Figure 2E). This result was consistent with our previous studies (Wang L et al., 2018; Chen et al., 2019; Wang et al., 2020). The IR780 encapsulation efficiency was 32.18%, and the drug-loading efficiency was 0.64%. Then, SOSG was applied to confirm the IR780 nanoparticles as a sonosensitizer based on the fluorescence intensity of the generated ^1^O_2_. As shown in Figure 2F, the fluorescence intensity of the SOSG solution (5 µM) containing IR780 nanoparticles (10 mg/ml) drastically increased 3-fold with prolonged irradiation within 210 s (2 W/cm^2^, 5 s). Next, the stability of the IR780@PLGA nanoparticles in 1×PBS or 10% FBS was monitored by Malvern size analysis (Supplementary Figure S1, see the Supporting Information). After 7 days, the size of the IR780@PLGA nanoparticles increased from 310 to 325 nm in PBS, and the IR780@PLGA nanoparticles also increased in size from 305 to 315 nm in FBS.

### *In Vitro* Antibacterial Activity Assay

**FIGURE 3:**
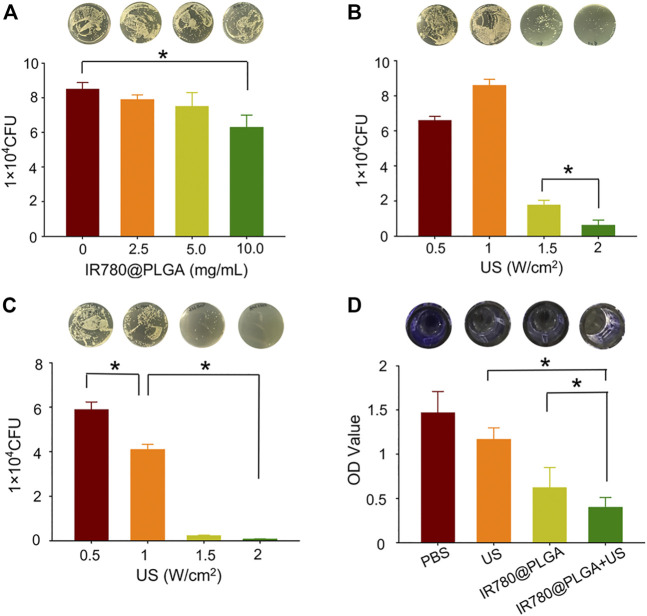
**(A)** Plate-counting results of MRSA bacteria after coculture with IR780@PLGA nanoparticles at different concentrations for 24 h. **(B)** Plate-counting results of MRSA bacteria after treated with US irradiation at different intensities. **(C)** Plate-counting results of MRSA bacteria after treated with the same concentration of IR780@PLGA nanoparticles (original concentration of 10 mg/ml) and different intensities of US irradiation. **(D)** Crystal violet staining results of the biofilm after different treatments for 36 h.

To evaluate the dose-dependent antibacterial capability of IR780@PLGA nanoparticles, MRSA bacteria were incubated with different concentrations of IR780@PLGA nanoparticles, and then the number of bacterial colonies was counted after culture at 37°C for 24 h. As shown in Figure 3A; Supplementary Figure S2 (see the Supporting Information), there were fewer bacterial colonies in the group of IR780@PLGA nanoparticles (100 μg/ml, with an initial concentration of 10 mg/ml) than those in the control group without nanoparticles intervention (*p* < 0.05). As the concentration of nanoparticles increased, the number of bacterial colonies gradually decreased, meaning that the IR780@PLGA nanoparticles have antibacterial capability and possess the dose-dependent properties.

To evaluate the US intensity–dependent antibacterial capability, MRSA bacteria were introduced to different intensities of interval US irradiation, and then the number of bacterial colonies was counted after culture at 37°C for 24 h. As shown in Figure 3B; Supplementary Figure S3 (see the Supporting Information), the number of bacterial colonies in the 2 W/cm^2^ US intensity group was obviously fewer than those in the other three groups (*p* < 0.05). Compared with the 0.5 W/cm^2^ US intensity group, the number of bacterial colonies in the 1 W/cm^2^ US intensity group showed a slight increase, which may be because low-intensity US irradiation can promote bacterial growth. In contrast, when the US intensity increased to 1.5 W/cm^2^, the number of bacterial colonies sharply decreased, and when the US intensity increased to 2.0 W/cm^2^, the bacteria were almost completely obliterated.

Subsequently, the SDT efficacy of IR780@PLGA nanoparticles was assessed. MRSA bacteria were incubated with the same concentration of IR780@PLGA nanoparticles (10.0 mg/ml) and introduced to different intensities of interval US irradiation. As shown in Figure 3C; Supplementary Figure S4 (see the Supporting Information), the number of bacterial colonies in the 1.5 W/cm^2^ and 2.0 W/cm^2^ of US intensity groups were almost killed, and they had obviously fewer bacterial numbers than the other two groups (*p* < 0.05), meaning the IR780@PLGA nanoparticles with the higher intensity of US irradiation have a remarkable antibacterial capability. Thus, we used the US intensity of 2.0 W/cm^2^ and nanoparticles concentration of 10 mg/ml in the following antibacterial experiments.

Subsequently, biofilms of MRSA bacteria were obtained to evaluate the bacterial biofilm destruction ability of IR780@PLGA nanoparticles by SDT. The crystal violet staining results of biofilms showed that both the US-irradiated and IR780@PLGA nanoparticles had antibiofilm capabilities compared with the control group, while the SDT group (IR780@PLGA nanoparticles + US group) had the best ability to destroy the bacterial biofilm compared with other three groups (Figure 3D), which indicates that the antibacterial effect of IR780@PLGA nanoparticles by SDT may be contributing to their antibiofilm capability.

**FIGURE 4:**
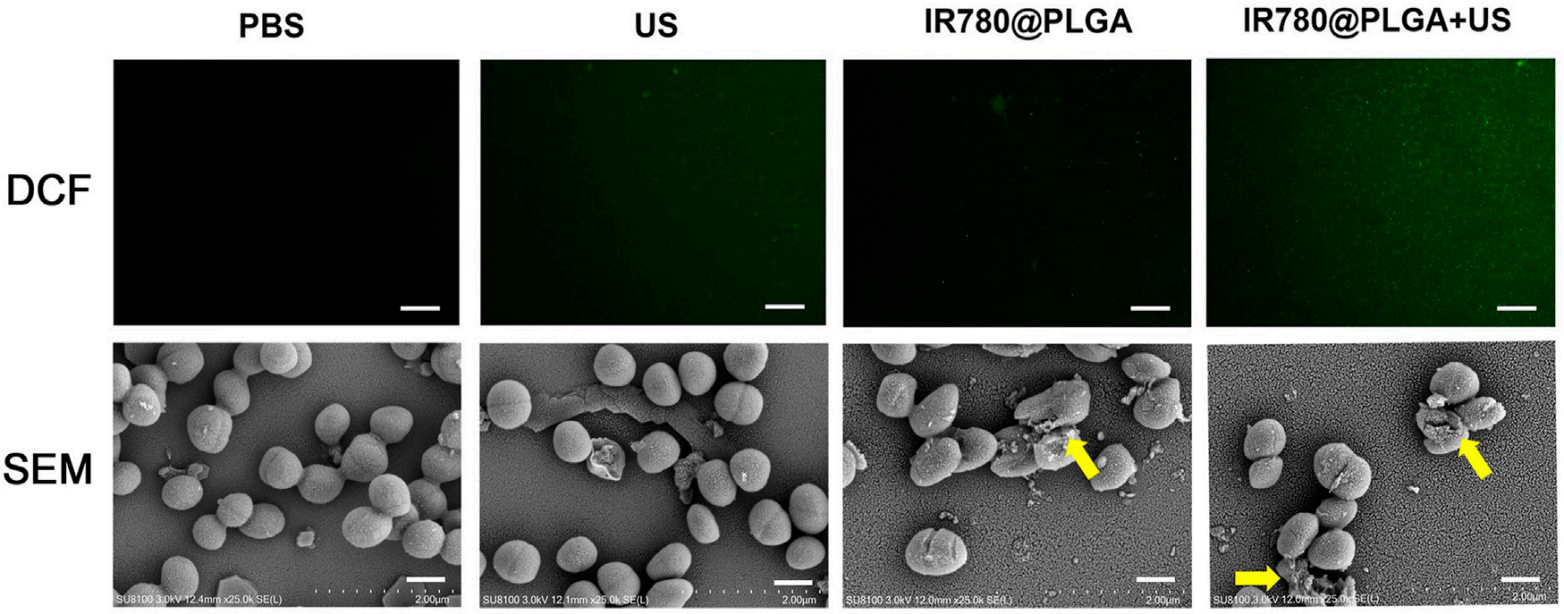
**(A)** Fluorescent images of DCF-stained ^1^O_2_ generation by bacteria at various treatments, scale bar = 50 μm. **(B)** SEM images of the bacterial biofilm at various treatments, scale bar = 5 μm.

Then, to explore the potential of IR780@PLGA nanoparticles as sonosensitizers in bacteria, DCFH-DA was used to detect ^1^O_2_ generation based on fluorescence intensity (DCF). As shown in Figure 4, MRSA bacteria treated with PBS, US irradiation only, or IR780@PLGA nanoparticles without US irradiation exhibited negligible fluorescence. In contrast, bacteria treated with IR780@PLGA nanoparticles with US irradiation displayed obvious green fluorescence, indicating that ^1^O_2_ was generated based on the sonosensitizer of IR780@PLGA nanoparticles exposed to US irradiation. For SEM image detection, biofilms remained intact in the PBS group and exhibited little destruction in the US group. With the treatment of IR780@PLGA nanoparticles, a part of the biofilm began to be eliminated and broke into pieces, especially with US irradiation, which is consistent with the results of the crystal violet staining described previously.

### *In Vivo* Imaging and Antibacterial Therapy for Bacterial Infections

**FIGURE 5:**
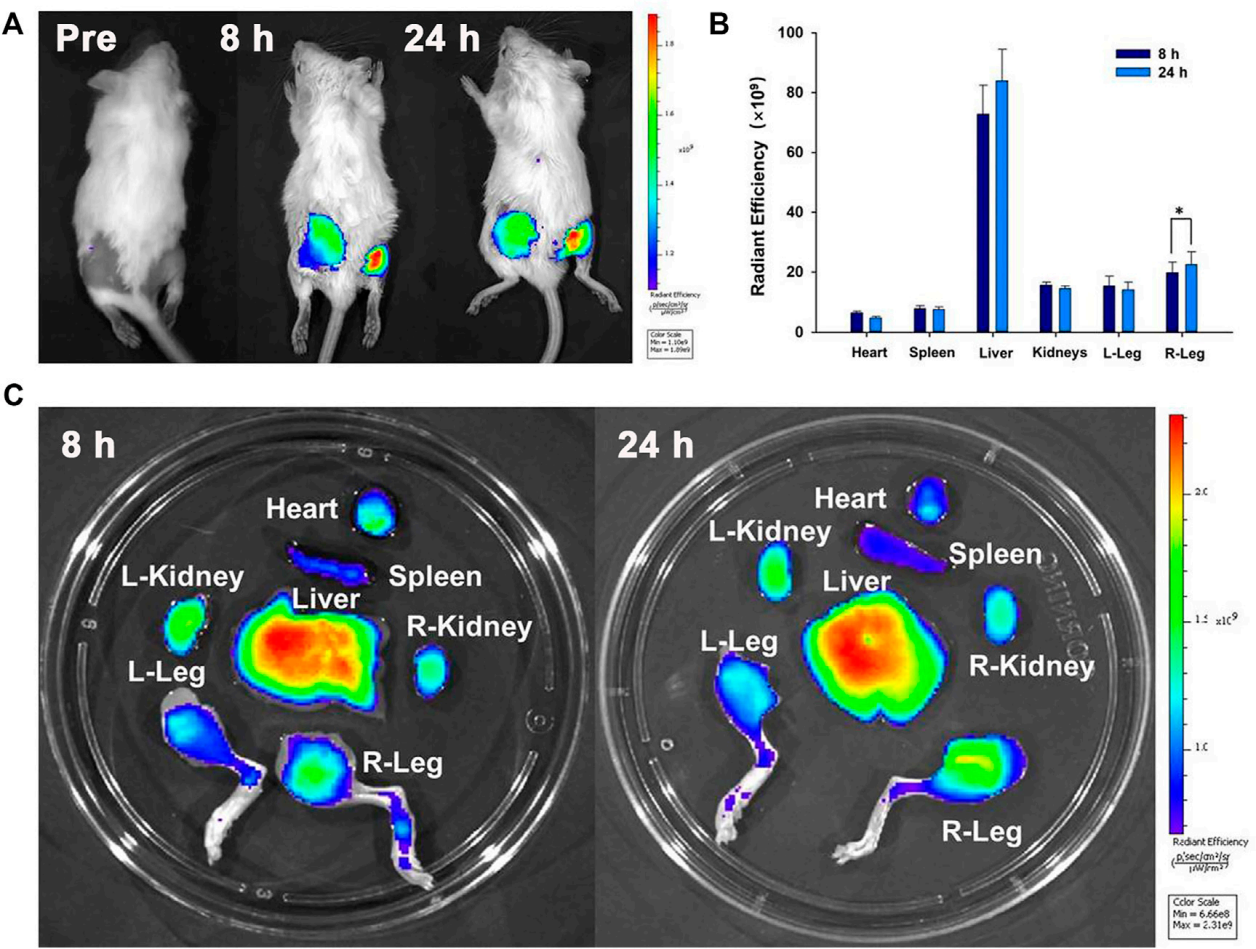
**(A)** Time-lapse NIR fluorescence images of MRSA-infected mice *in vivo* after injection of IR780@PLGA nanoparticles. **(B)** Averaged *ex vivo* NIR fluorescence intensities and **(C)** NIR fluorescence images of major organs and legs in MRSA-infected mice; the fluorescence of the right leg 24 h postinjection was significantly higher than that at 8 h postinjection (*p < 0.05).

To confirm that the nanoparticles could reach the bacterial infection, the NIR fluorescence imaging performance of IR780@PLGA nanoparticles was measured. After injection of nanoparticles for 8 h or 24 h, obvious fluorescence was observed at the infected sites of mice (Figure 5A). The fluorescence of the right leg (MRSA-infected site) was obviously higher than that of the left leg (healthy leg) at both time points, which may be attributed to potent synergism of the enhanced permeation and retention (EPR) effect at the infection site (Azzopardi et al., 2013; Maeda, 2013). Moreover, as shown in Figures 5B,C, the fluorescence of the right leg 24 h postinjection was significantly higher than that at 8 h postinjection (*p* < 0.05); therefore, in this study, the best therapeutic time for exposure to US irradiation was 24 h postinjection of nanoparticles.

**FIGURE 6:**
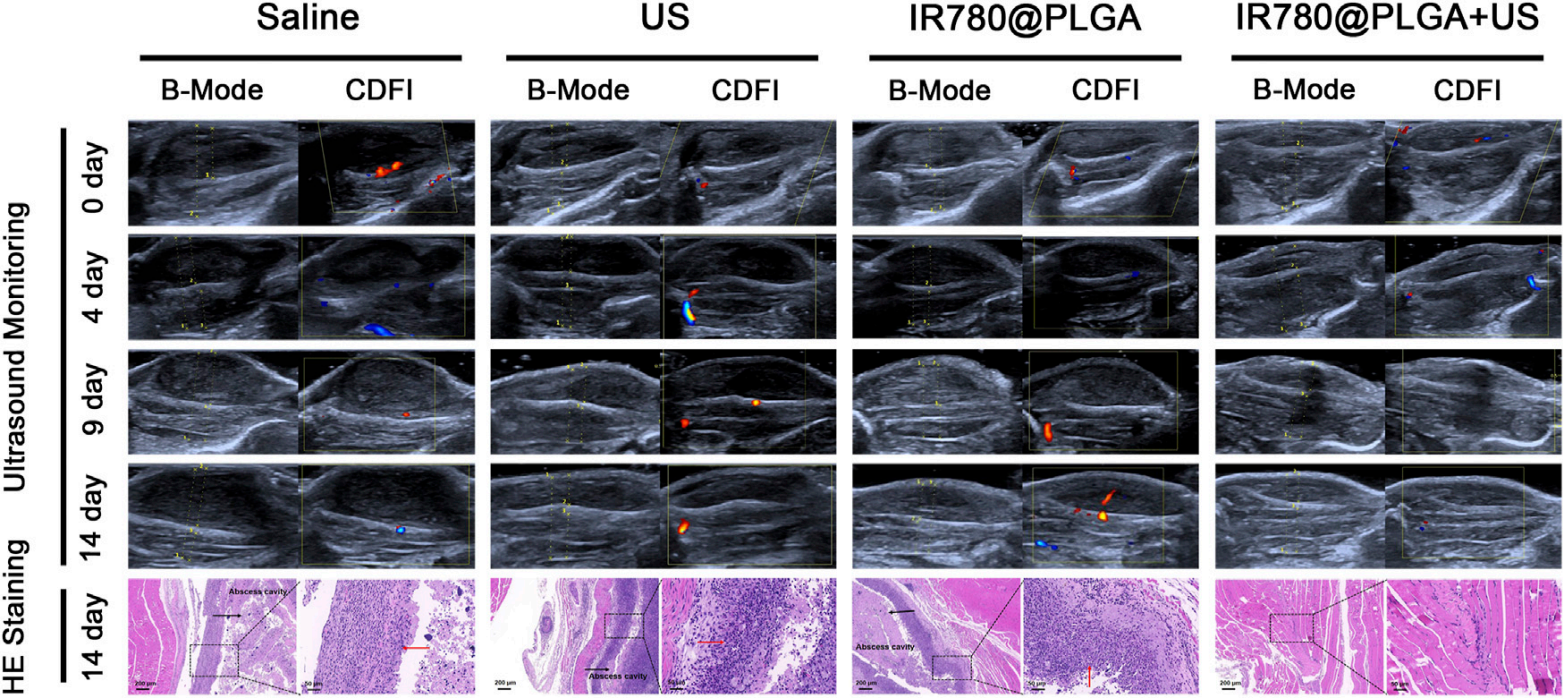
Representative US images and histological images of the MRSA-infected mice within 14 days postinjection in different groups. The arrows in histological images indicate the inflammatory exudate and necrotic tissue.

**FIGURE 7:**
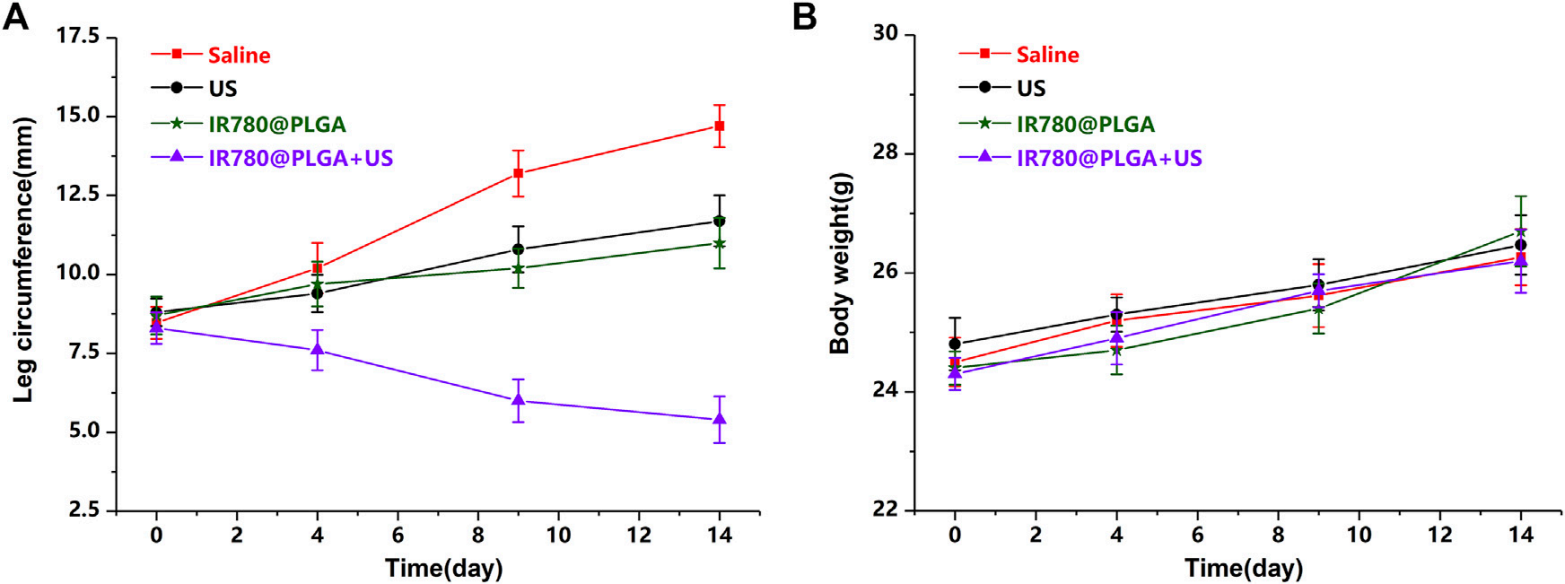
**(A)** Average leg circumference curve measured by US and **(B)** average body weights of mice after different treatments (*p < 0.05).

To better monitor the therapeutic efficacy of deep-seated infection, a US apparatus was introduced to visualize the therapeutic progression of nanoparticles-mediated SDT. Figure 6 shows representative images of US (including B- and CDFI-mode US) from different treatment groups during observation, and the corresponding quantitative analysis of infected leg circumference measured by US at different time points is shown in Figure 7A. Diagnostic US has a noninvasive, inexpensive, and fast detection nature, and this high-frequency portable US can be brought into the animal room to monitor the inflammation progression of mice more accurately in the barrier system to reduce the limitation that animals have to be brought out of the animal room for monitoring by other methods (Pang et al., 2019a; Pang et al., 2019b; Wang et al., 2021). B-mode US could directly observe the leg size and echogenicity change of the infected legs, allowing measurements of the quantitative changes in the antibacterial therapy in different treatment groups. CDFI-mode US could offer the blood flow signals of the infected lesion. A richer blood flow signal usually indicates more severe inflammation. As shown in Figures 6, 7A, the leg size in the IR780@PLGA + US group was significantly smaller than that in the other three groups, similar to the blood flow signals. Treatment with saline had no effect on inhibiting MRSA infection, presenting significant deterioration from diffuse muscular edema and focal liquefactive necrosis, and an obvious abscess cavity was generated on histopathology. While US alone or nanoparticles alone slightly alleviated the infection, abscess cavities were also observed by HE staining. In contrast, when nanoparticles were combined with US irradiation, MRSA infection significantly suppressed mild inflammatory cell infiltration. These results implied that IR780@PLGA nanoparticles-mediated SDT has remarkable antibacterial efficacy. As shown in Figure 7B, the body weight curves relative to time after different treatments showed no significant difference among these groups and no obvious weight loss, indicating the good biosafety of these treatments.

### *In Vivo* Toxicity Study

**FIGURE 8:**
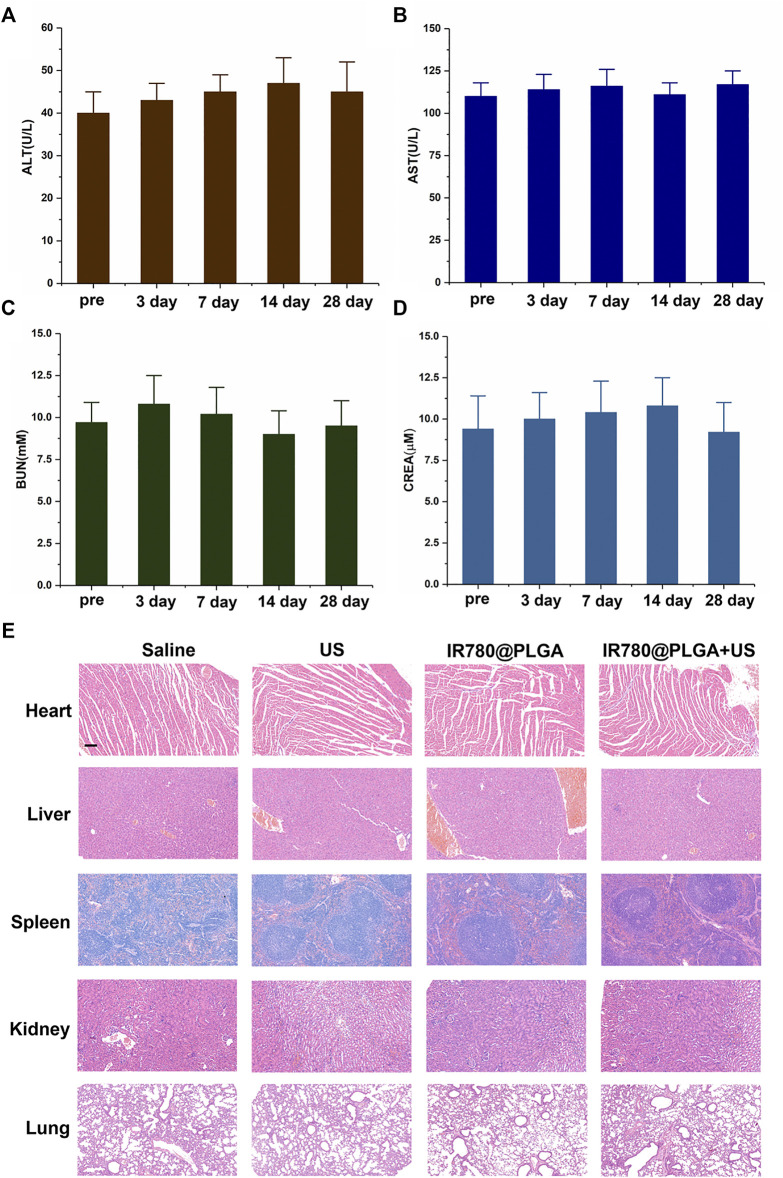
*In vivo* toxicity study. Serum biochemistry assays including **(A)** ALT, **(B)** AST, **(C)** BUN, and **(D)** CREA were assessed at 3, 7, 14, and 28 days after injection of nanoparticles; the preinjection of the nanoparticles group was used as a control; the levels of these four indicators were all within normal ranges at different time points (*p* > 0.05). **(E)** H&E staining sections of major organs were harvested at 28 days at different treatments, scale bar = 50 μm. No noticeable abnormality was observed in the heart, liver, spleen, lung, and/or kidney.

To assess the *in vivo* toxicity of the IR780@PLGA nanoparticles, serum biochemistry assays including ALT, AST, BUN, and CREA were measured in blood samples from healthy nanoparticles-treated mice. As shown in Figures 8A–D, the levels of these four indicators were all within normal ranges at different time points. Furthermore, major organs of the MRSA-infected mice after different treatments at 28 days were harvested and stained with H&E for histological analysis (Figure 8E). No noticeable organ damage was observed in H&E-stained sections of the major organs, indicating that the IR780@PLGA nanoparticles have good biosafety.

## Conclusion

In conclusion, we first reported IR780-based PLGA nanoparticles for noninvasive US-monitored antibacterial SDT of MRSA infection. High-frequency diagnostic US was introduced to monitor the sonotherapeutic progression of bacterial myositis by therapeutic low-frequency US. Importantly, the *in vitro* and *in vivo* results confirmed that the IR780@PLGA nanoparticles combined with US irradiation possess high efficiency for antibacterial therapy. Therefore, the IR780@PLGA nanoparticles could be applied as useful antibacterial SDT sonosensitizers and could be used to enhance the antibacterial efficacy against MDR bacterial infection.

## Data Availability

The raw data supporting the conclusion of this article will be made available by the authors, without undue reservation.
