# Supplementary material for: IR780 Based Sonotherapeutic Nanoparticles to Combat Multidrug-Resistant Bacterial Infections
Source: Front Chem. 2022 Jan 24;10:840598. doi: 10.3389/fchem.2022.840598 (PMC8818736; doi:10.3389/fchem.2022.840598)
Supplement: Supplementary file 1 [file DataSheet1.docx]

IR780 based sonotherapeutic nanoparticles to combat multidrug-resistant bacterial infections

Biying Huang **^1,2 #^**, Long Wang **^3,4,5,6 #^**, Kui Tang **^1,2^**, Sijie Chen **^1,2^**, Yan Xu **^1,2^**, Haiqin Liao **^1,2^**, Chengcheng Niu **^1,2^** *

**^1^** Department of Ultrasound Diagnosis, The Second Xiangya Hospital, Central South University, Changsha, Hunan 410011, China

**^2^** Research Center of Ultrasonography, The Second Xiangya Hospital, Central South University, Changsha, Hunan 410011, China

**^3^** Department of Orthopedics, Xiangya Hospital, Central South University, Changsha, Hunan 410008, China

^4^ Hunan Engineering Research Center of Biomedical Metal and Ceramic Implants, Xiangya Hospital, Central South University, Changsha, China

^5^ National Clinical Research Center for Geriatric Disorders, Xiangya Hospital, Central South University, Changsha, China

^6^ Hunan key laboratary of aging biology, Xiangya Hospital, Central South University, Changsha, China

**Address all correspondence to:** Chengcheng Niu, Department of Ultrasound Diagnosis and Research Center of Ultrasonography, The Second Xiangya Hospital, Central South University, Changsha, Hunan, China, 410011; E-mail: [niuchengcheng@csu.edu.cn](mailto:niuchengcheng@csu.edu.cn)

^#^ These authors have contributed equally to this work.

Figures


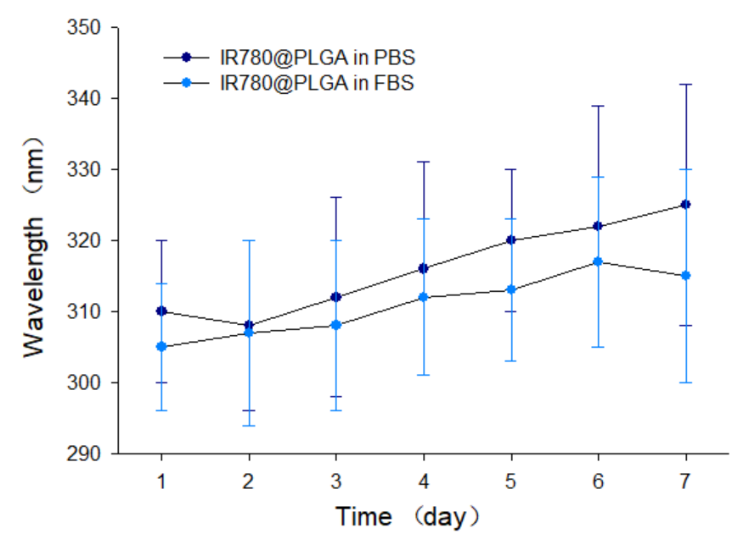


Figure S1. Size distributions of IR780@PLGA nanoparticles in 1 × PBS or 10% FBS in 7 days.


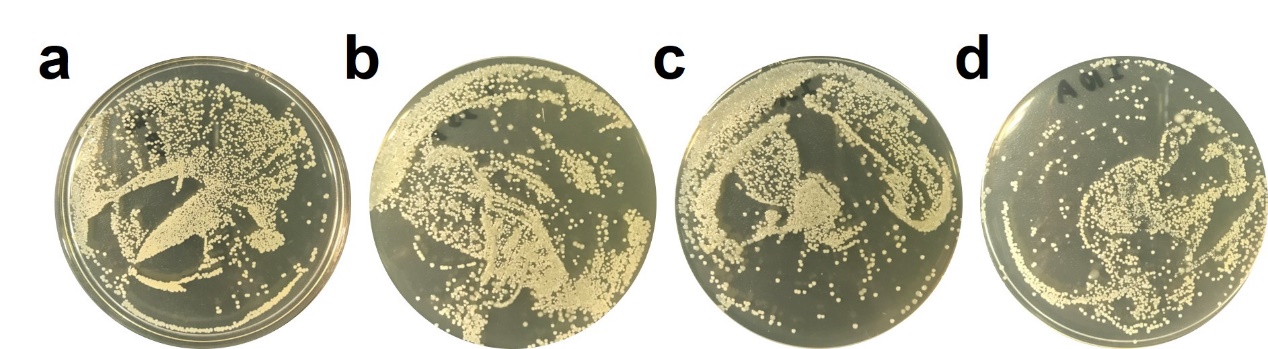


Figure S2. Plate counting results of MRSA bacteria after coculture with IR780@PLGA nanoparticles at different concentrations for 24h: (a) 0 mg/mL; (b) 2.5 mg/mL; (c) 5.0 mg/mL; (d) 10.0 mg/mL.


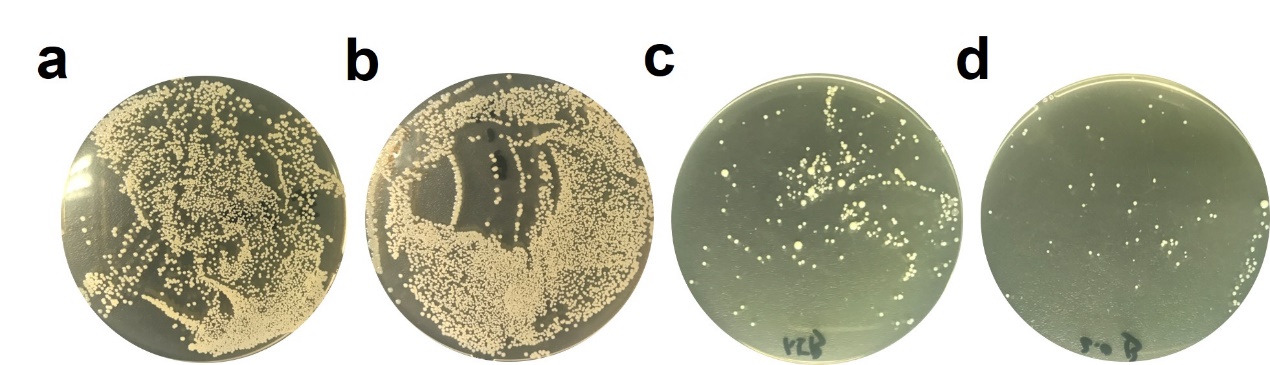


Figure S3. Plate counting results of MRSA bacteria after treated with US irradiation at different intensities: (a) 0.5 W/cm^2^; (b) 1 W/cm^2^; (c) 1.5 W/cm^2^; (d) 2 W/cm^2^.


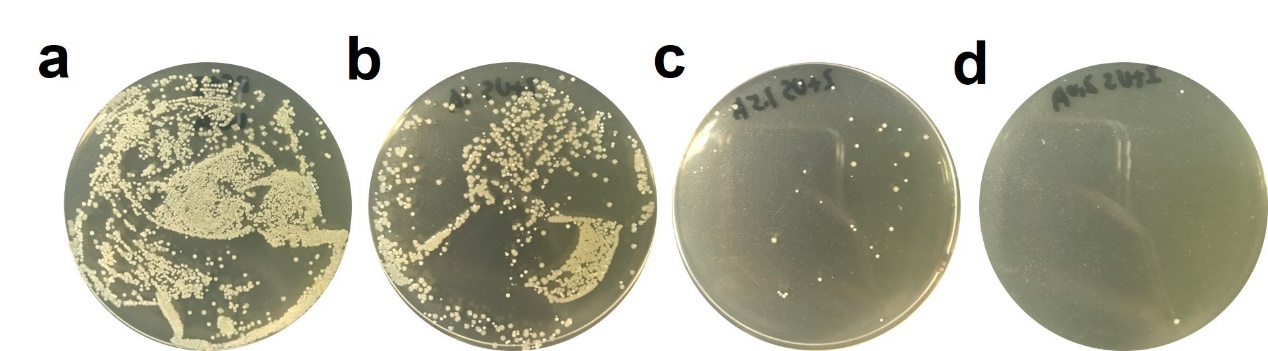


Figure S4. Plate counting results of MRSA bacteria after treated with the same concentration of IR780@PLGA nanoparticles (original concentration of 10 mg/mL) and different intensities of US irradiation: (a) 0.5 W/cm^2^; (b) 1 W/cm^2^; (c) 1.5 W/cm^2^; (d) 2 W/cm^2^.
